# Maceration in White Winemaking: Enhancing Phenolics, Volatile Aromas and Sensory Characteristics of Chardonnay and Italian Riesling Wines

**DOI:** 10.3390/molecules31132223

**Published:** 2026-06-24

**Authors:** Weina Xu, Qingxin Yan, Yi He, Xiaohui Sun, Jicheng Zhan, Weidong Huang, Guangli Xia

**Affiliations:** 1 School of Pharmacy, Shandong Medical and Pharmaceutical University, Yantai 264003, China; 2 Yantai Institute of China Agricultural University, China Agricultural University, Yantai 264670, China; 3 Shandong Academy of Grape, Shandong Academy of Agricultural Sciences, Jinan 250100, China

**Keywords:** cold maceration, skin-contact fermentation, Chardonnay, Italian Riesling, white wine, phenolics, volatile aroma, sensory evaluation

## Abstract

Maceration techniques like cold maceration before fermentation (CM) and skin-contact fermentation (SF) are widely used in winemaking. However, their application remains limited in white winemaking, representing an important objective for the production of diverse white wines. This study systematically investigated the impacts of CM and SF on phenolics, volatile aromas and sensory properties of Chardonnay and Italian Riesling wines in Yantai wine region. Both CM and SF significantly increased the total phenolic content, especially with gallic acid and quercitrin contents rising 11.65- and 10.02-fold in Chardonnay, as well as catechin and quercitrin increasing 9.05- and 10.82-fold in Italian Riesling under 100% SF. Moreover, 72 h CM and 100% SF showed higher volatile aroma contents in both wines compared to other CM and SF treatments. Esters, including ethyl octanoate, ethyl hexanoate and isoamyl acetate, contributed to the improved floral and fruity aromas in maceration-treated Chardonnay wines, whereas esters and terpenes drove the aromatic profile of Italian Riesling wines, with terpenes rising 1.67- and 3.27-fold after CM and SF. The two varieties differed in wine color, with Italian Riesling wines displaying a stronger yellow hue and reduced lightness under 72 h CM, 50% SF and 100% SF. Sensory evaluation by a panel containing seven trained assessors found that SF-treated wines exhibited more intense flavor and balanced taste and CM-treated wines showed more freshness. These findings provide theoretical support for tailored maceration to enhance varietal expression in the specific region and diversify white wine production.

## 1. Introduction

The white wine market is evolving towards diversification and high-quality, driven by emerging consumer preferences [1,2]. Traditional white wine production generally involves fermenting grape juice at 15–20 °C, resulting in low levels of phenolics and volatile compounds in wines [3]. In recent years, various skin maceration techniques such as cold maceration before fermentation (CM), skin-contact fermentation (SF) and carbonic maceration have been increasingly utilized in winemaking to enhance wine quality, especially for red wine [4,5,6].

CM is commonly used in red winemaking, but less so in whites. This technique involves contact between the skins, seeds and grape juice at low temperatures prior to alcoholic fermentation [5,7]. Studies have shown that CM has positive impacts on Sauvignon Blanc, Arneis and Chardonnay white wines, among others [7,8,9]. This process facilitates the extraction of phenolics, aroma compounds and various nutrients from grape skins and seeds, thereby accelerating fermentation onset and promoting flavor development [9,10,11,12]. Maceration duration is the primary factor, showing a linear correlation with phenolic content [5,12]. SF, another maceration technique involving controlled skin contact during alcoholic fermentation, is also well-documented in red winemaking [11,13]. Proper SF application elevates the phenolic content, aroma complexity and mouthfeel structure of wines [14]. Notably, excessive phenolic extraction may introduce undesirable astringency, bitterness and color changes in white wines [15]. To date, only limited studies, such as Prezioso et al. have examined the influence SF on the phenolic composition, volatile profile and sensory properties of Minutolo and Verdeca white wines [12]. In general, these maceration technologies for white wines have received far less research attention than those for red wines. More efficient techniques are thus in demand for white wine production. In addition, most previous studies focus solely on either CM or SF [8,12], with few comparisons clarifying how the two techniques differ in modulating wine quality. Furthermore, current research on maceration applications in white wine production has focused on only a limited number of grape varieties. The effects of maceration techniques on oenological characteristics cannot be generalized, owing to multiple enological factors including grape varietals and maturity indices [9,16,17,18]. Therefore, further investigation is needed to determine whether different white grape varieties yield inconsistent compositional and sensory outcomes when subjected to the same CM and SF treatments.

Chardonnay and Italian Riesling are the two crucial white grape varieties in Yantai wine appellation, which is a representative wine-producing area in China. Both varieties exhibit excellent enological potential, making them particularly valuable for local winemaking [19,20]. Chardonnay and Italian Riesling white wines produced by the traditional juice fermentation exhibit aromatic profiles dominated by refreshing floral, honey and fruity notes, but often lack intensity and complexity, limiting terroir expression [7]. Thus, investigating the effects of different maceration treatments and optimizing winemaking practices for Chardonnay and Italian Riesling can highlight varietal differences and enhance white wine typicity and diversity.

In this study, Chardonnay and Italian Riesling grapes from the Yantai wine appellation were subjected to CM treatments for 24 h, 48 h, and 72 h and SF treatments with 25%, 50% and 100% grape solids (skins and seeds), with each treatment performed in triplicate. By comprehensive analysis of the chemical and sensory profiles of wines, we systematically investigated the effects of the two maceration treatments on wine quality. To our knowledge, this is the first study to investigate the effect of skin-contact macerations on Italian Riesling and to employ a multidimensional analysis of how two different maceration techniques affect two different white grape varieties. This study advances the understanding of how maceration strategies can be optimized to enhance varietal expression and diversify white wine profiles. It will provide valuable practical and region-specific guidance for winemakers to refine their winemaking practices, especially for Chardonnay and Italian Riesling, and to produce white wines that cater to the diverse preferences of wine consumers.

## 2. Results and Discussion

### 2.1. Fermentation Process Analysis

**Figure 1:**
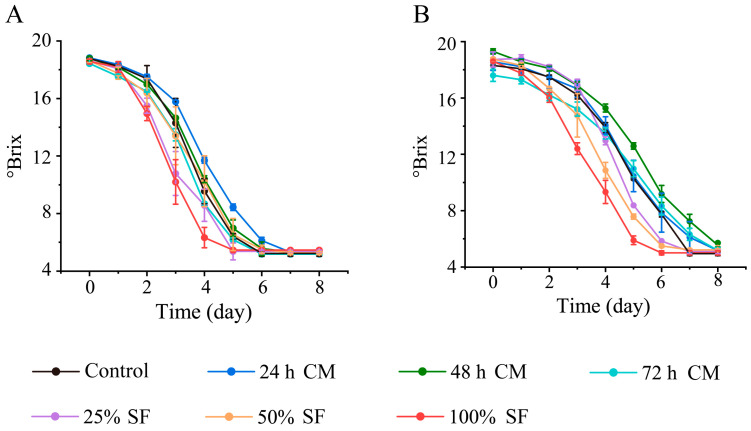
Effect of different maceration processes on ^°^Brix value during Chardonnay (**A**) and Italian Riesling (**B**) white wine fermentation. CM, cold maceration before fermentation. SF, skin-contact fermentation.

Total soluble solid analysis revealed that all maceration treatments enabled complete fermentation for both Chardonnay and Italian Riesling wines, but altered the fermentation kinetics (Figure 1A,B). For Chardonnay, SF treatments significantly increased the fermentation rate from the second day compared to the control, with 25% and 100% SF shortening fermentation time by one day (Figure 1A). Similarly, SF treatments significantly accelerated fermentation rate and reduced fermentation duration of Italian Riesling, with these improvements becoming more pronounced as the skin addition increased (Figure 1B). This promoting effect was consistent with the previous study by Sancho-Galán et al. [21]. It may be attributed to the available nutrients and co-factors from grape skins and seeds entering the fermentation broth due to SF, which promoted yeast growth and proliferation [21,22]. In addition, the extension of fermentation duration by CM in both varieties, especially in Italian Riesling (Figure 1A), has not been reported previously. It is hypothesized that this delay may be related to the leaching of inhibitory compounds such as stilbenes and phenolics under low-temperature conditions [21,23].

### 2.2. Basic Physicochemical Parameter Analysis

The physicochemical characteristics of Chardonnay and Italian Riesling white wines are presented in Table 1. SF treatments considerably enhanced the residual sugars in both Chardonnay and Italian Riesling white wines (Table 1). Grape skins have been reported to contain substantial amounts of sugars such as glucose, fructose, arabinose, and xylose, among others [24]. It is possible that the presence of non-fermentable pentoses derived from grape skins, together with skin-embedded sugars, contributed to elevated residual sugar levels in SF wines. Total acid content decreased significantly in all maceration treatments of Chardonnay, especially in 24 h CM and 25% SF, with a corresponding rise in pH (Table 1). A similar trend was observed in Italian Riesling white wines, which exhibited reduced acid content under SF treatments and elevated pH in all maceration groups (Table 1). It has been reported that grape skins are rich in ions, which can be extracted from grape solids into the liquid during skin-contact maceration [8]. Among these ions, potassium ions can combine with tartaric acid to form sparingly soluble potassium bitartrates, which precipitates during fermentation [1,6,8]. Thus, free acid was removed from the wine, leading to a decrease in total acid and a consequent increase in pH. In addition, Chardonnay wines showed a lower pH compared with Italian Riesling wines. This difference is likely related to intrinsic varietal factors such as the content and composition of organic acids. Volatile acid, closely associated with wine oxidation and quality, should be controlled within certain limits to avoid negative effects [25]. In this study, SF and CM with short-time markedly reduced volatile acid content in both wines (Table 1), aligning with the findings of Palomo et al. for Albillo wines [26]. With the prolongation of CM, the content of volatile acid showed an upward trend. Tannin contents increased in both varieties following CM and SF treatments (Table 1), which can be attributed to the concentrated tannin in grape skins and the enhanced extraction with extended skin contact [27,28].

### 2.3. Phenolics Compounds Analysis

Phenolics, important secondary metabolites in grapes, are closely related to wine flavor and antioxidant properties [29]. In this study, the total phenolic content exhibited a significant increase in both Chardonnay and Italian Riesling wines with prolonged CM duration and higher skins addition of SF, with rises of 13.28% under 72 h CM and 41.28% under 100% SF in Chardonnay wine, and 9.92% under 72 h CM and 25.09% under 100% SF in Italian Riesling wine (Table 1). The more pronounced effect was observed in SF treatments, which suggested that phenolic compounds are effectively extracted during alcoholic fermentation, facilitated by the breakdown of the pectin layer in grape cell wall [30,31].

**Figure 2:**
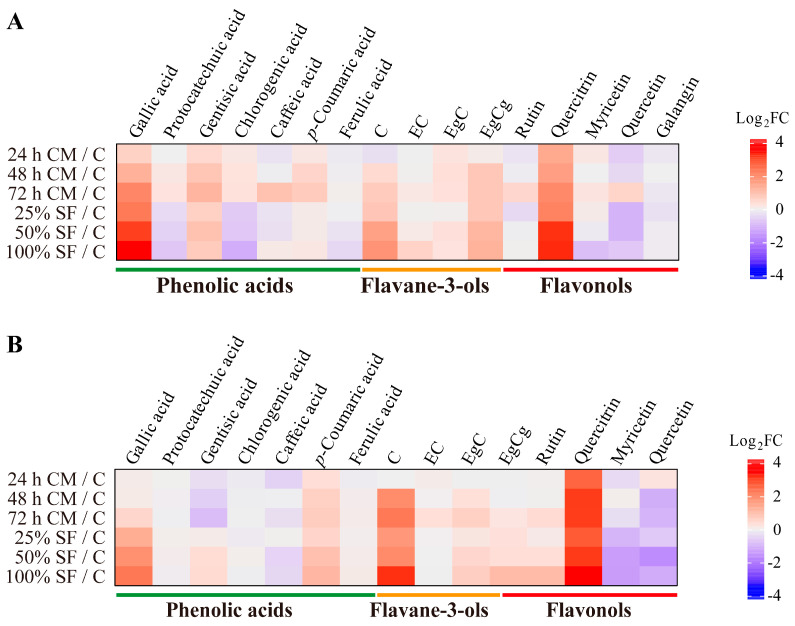
Heat map based on the concentrations of phenolics in Chardonnay (**A**) and Italian Riesling (**B**) white wines under different maceration processes. Group: C, control. CM, cold maceration before fermentation. SF, skin-contact fermentation. Phenolic compound: C, catechin. EC, epicatechin. EgC, epigallocatechin. EgCg, epigallocatechin gallate.

We then investigated various phenolic compounds including seven phenolic acids, four flavane-3-ols and five flavonols in the two wines under different treatments (Table S1). In Chardonnay, CM significantly enhanced the contents of gallic acid, gentisic acid and *p*-coumaric acid, with gallic acid exhibiting a notable 4.17-fold increase after 72 h (Figure 2A and Table S1). SF treatments substantially increased gallic acid (4.93- to 11.65-fold) and gentisic acid (1.54- to 1.93-fold) contents in Chardonnay wines (Figure 2A and Table S1). In Italian Riesling, CM also resulted in an obvious increase in gallic acid and *p*-coumaric acid, and SF induced more pronounced gallic acid accumulation (Figure 2B and Table S1). Gallic acid exists in free, esterified and complexed forms, and it can be released via direct extraction or hydrolysis of seed-derived epigallocatechin gallate (EgCg) [14,28]. Maceration treatments likely facilitate the release and extraction of gallic acid during skin contact, particularly in the presence of alcohol, thereby increasing its concentration in wines [32].

Flavane-3-ols provide bitterness and astringency in wines [33]. Among four detected flavane-3-ols, epigallocatechin (EgC) exhibited the highest content, followed by catechin (C), epicatechin (EC) and EgCg (Table S1). Both CM and SF treatments enhanced the total flavane-3-ols concentrations in Chardonnay and Italian Riesling wines (Table S1). Prolonged CM and SF with higher skins addition significantly boosted C levels in both Chardonnay and Italian Riesling wines, with other flavane-3-ols exhibiting more moderate increases (Figure 2A,B). The most pronounced enhancement was observed in Italian Riesling wines, where the 72 h CM and 100% SF treatments increased C content by 4.7-fold and 9.05-fold, respectively (Figure 2B). This notable rise may be attributed to the facilitated migration of C during maceration, a process consistent with the mechanism reported by Di Lecce et al. [34].

Flavonols, mainly from grape skins, are crucial for imparting astringency and antioxidant properties to wines [35]. CM and SF treatments significantly enhanced the quercitrin content in both Chardonnay wines and Italian Riesling wines, representing increases of 2.73- to 10.02-fold and 5.87- to 10.82-fold, respectively (Table S1, Figure 2A,B), suggesting that CM and SF can effectively facilitate the extraction of quercetin from the grape’s pericarp and seeds. In addition, maceration treatment reduced the quercetin content in the two wines and myricetin content in Italian Riesling (Figure 2A,B). Previous studies have reported that some wild yeasts can catabolize quercetin and the yeast cell wall can absorb phenolics such as quercetin [36,37]. It is possible that the metabolic degradation and adsorption by wild yeast from grape skins led to the observed decreases.

**Figure 3:**
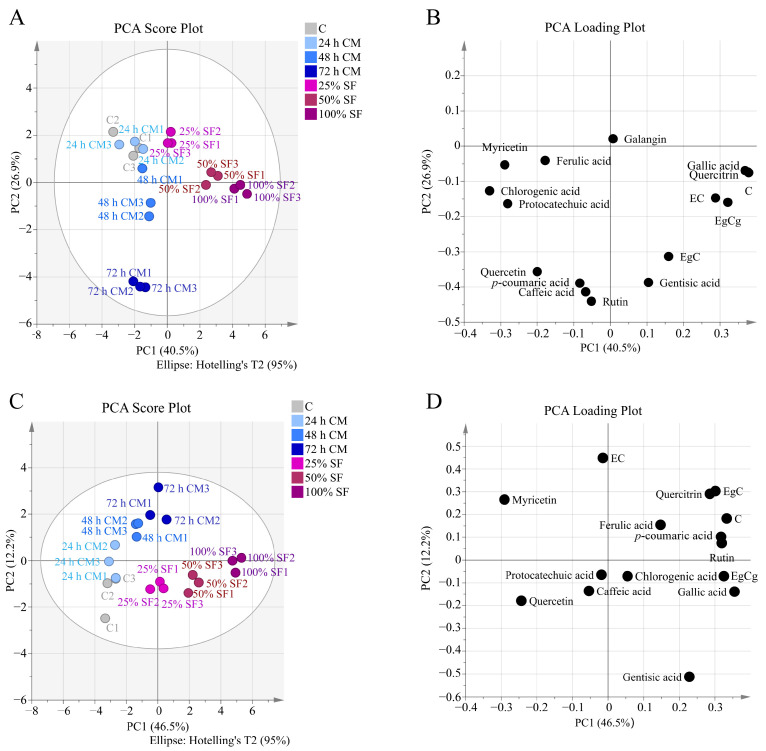
PCA analysis of the phenolic compounds in Chardonnay (**A**,**B**) and Italian Riesling (**C**,**D**) white wines. (**A**,**C**) are PCA score plot. (**B**,**D**) are PCA loading plot. Group: C, control; CM, cold maceration before fermentation; SF, skin-contact fermentation. Phenolic compound: C, catechin. EC, epicatechin. EgC, epigallocatechin. EgCg, epigallocatechin gallate.

Principal component analysis (PCA) was employed to facilitate a more intuitive comparison between different maceration in two wines. A distinct separation was observed between Chardonnay wines treated by CM and SF, with SF clustered on the positive axis of PC1 and CM clustered on the negative axis (Figure 3A), suggesting substantial differences in phenolic compounds induced by the two maceration methods. The 24 h CM treatment group clustered closely with the control, implying that short-duration cold maceration had only a minor influence on phenolic compounds (Figure 3A). The clear separation among different CM duration further demonstrated that maceration time significantly influences phenolic profiles of the wines (Figure 3A). Loading plot analysis revealed that gallic acid, C and quercitrin were positioned on the positive side of PC1-axis and correlated positively with the 100% SF treatment (Figure 3B), consistent with the result observed in heat map (Figure 2A). In Italian Riesling wines, distinct separations were observed among different treatments in the PCA score plot (Figure 3C). Moreover, the CM treatments were located in the positive side of PC1 and the negative side of PC2, whereas the SF treatments showed the opposite distribution (Figure 3C), indicating that CM and SF had different impacts on phenolic compounds in Italian Riesling wines. In the loading plot, gallic acid, EgCg, rutin and other compounds were positively correlated with SF treatments with high skin additions, in contrast to quercetin, which exhibited a negative correlation (Figure 3D).

Overall, both CM and SF significantly alter the phenolic profiles of the wines, with the exception of 24 h CM treatment in Chardonnay. These alterations of phenolics were highly dependent on the maceration method, duration and skin-contact level. Such pronounced changes in phenolics may exert both positive and negative influences on wine color properties and sensory characteristics. The relevant impacts on wine quality are further discussed in the subsequent sections.

### 2.4. Volatile Aroma Compounds Analysis by HS-SPME-GC-MS

Aroma is an important organoleptic characteristic of wine, which can directly determine the wine flavor [14]. Volatile aroma components in Chardonnay and Italian Riesling wines with different maceration treatments were detected by headspace solid-phase microextraction–gas chromatography–mass spectrometry (HS-SPME-GC-MS).

**Figure 4:**
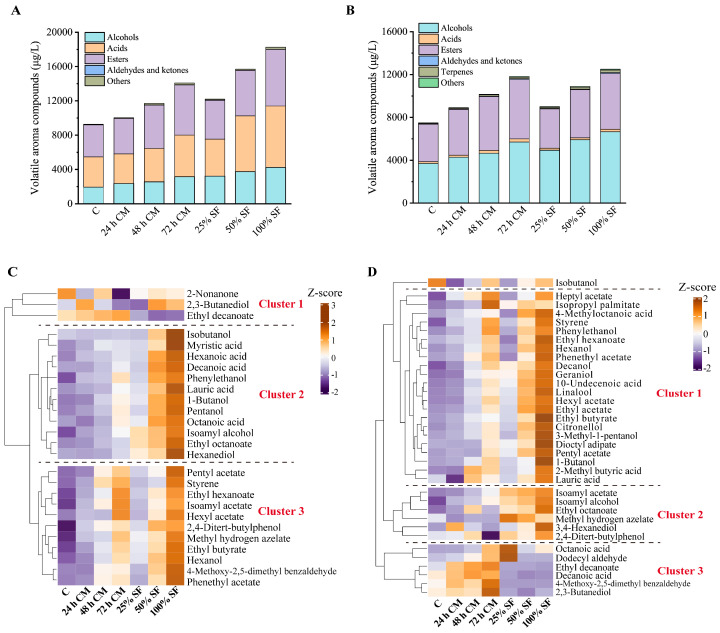
Volatile aroma compounds in Chardonnay and Italian Riesling white wines with different maceration processes. (**A**,**B**) Content of volatile aroma compounds in Chardonnay (**A**) and Italian Riesling (**B**) white wines. (**C**,**D**) Heat maps based on the concentrations of volatile aroma compounds in Chardonnay (**C**) and Italian Riesling (**D**) white wines. C, control. CM, cold maceration before fermentation. SF, skin-contact fermentation.

In Chardonnay wines, a total of 26 aroma compounds, including nine esters, eight alcohols, five acids, two aldehydes and ketones, and two other compounds, were identified, among which esters and acids were the most abundant (Table S2). The concentrations, odor sensory descriptors as well as odor thresholds (OTs) of identified aroma compounds are shown in Table S2. Esters, alcohols and acids were abundant in content and variety, and were the main source of aroma in Chardonnay white wine (Figure 4A and Table S3). Among different maceration treatments, the highest content of volatile aroma compounds was found in the wines fermented with 100% SF, which was about 1.95 times higher than that of control, followed by 50% SF and 72 h CM (Table S2). In Italian Riesling wines, a total of 35 aroma compounds, including 13 esters, nine alcohols, six acids, two aldehydes and ketones, three terpenes, and two other compounds, were detected. Esters and alcohols are the two most predominant types of volatile aroma compounds in terms of both concentration and variety, but the acid content was lower compared to Chardonnay wines (Figure 4B and Table S3). Both CM and SF treatments greatly increased the concentration of total volatile aroma compounds, with the most pronounced effect observed in 100% SF and 72 h CM treatments, which were 1.66- and 1.58-fold higher than the control, respectively (Table S3). These results revealed that CM and SF treatments significantly enhanced the aroma compound levels in both white wines. Moreover, longer maceration durations and higher skin ratios tended to further increase these levels. This enhancement, consistent with prior studies, is likely to influence wine aromatic intensity and complexity, thereby shaping the overall flavor profile of wine [8,14].

Aroma compounds in Chardonnay and Italian Riesling wines with different maceration treatments were further visualized using clustered heat maps (Figure 4C,D). Cluster analysis revealed that aroma compounds in Chardonnay wines were classified into three different clusters. SF treatments, especially those with high grape skin additions (50% and 100%) significantly increased the concentration of volatile compounds in cluster 2 (Figure 4C). This cluster included most alcohols and all acids in Chardonnay wines, such as isoamyl alcohol, phenylethanol, decanoic acid and hexanoic acid (Figure 4C). Among them, isoamyl alcohol (1076.14–2567.22 μg/L) and phenylethanol (459.73–802.20 μg/L) were the most abundant alcohols, and pentanol exhibited the most pronounced increase under 50% and 100% SF treatments, with concentrations rising 3.91-fold and 4.83-fold, respectively. In addition, 100% SF treatment increased the acid concentration by 2.03-fold in Chardonnay wine, potentially contributing to cheese, fatty characters in wines [13]. Alcohols and acids in wines are mainly generated as byproducts of amino acid and fatty acid metabolism during alcoholic fermentation, respectively. Their production is highly sensitive to several factors such as available assimilable nitrogen and temperature, as well as suspended solid content, which is modulated by the presence of grape solids during fermentation in SF treatment [38]. In addition, ethyl octanoate was classified in cluster 2, which had high odor activity values (OAVs), likely contributing to its fruity, sweet and waxy notes [8]. In cluster 3, prolonged CM and the SF with more skin additions increased the concentration of aroma compounds in Chardonnay wines (Figure 4C). Most of the esters (7/9), which confer floral and fruity aromas to the wines [18], were grouped into this cluster. Esters including ethyl hexanoate (751.98–1327.21 μg/L), isoamyl acetate (168.17–334.82 μg/L), and ethyl butyrate (751.98–1320.17 μg/L), exhibited OAVs exceeding 1, with concentrations highest under the 100% SF treatment, followed by 72 h CM. These esters may directly or through synergistic contributions to the fruity and floral aromas of Chardonnay [19].

Volatile aroma compounds in Italian Riesling wines were also classified into three clusters (Figure 4D). Cluster 1 contained nine esters, five alcohols, four acids, three terpenes and styrene. The concentrations of these aromas significantly increased with the prolonged CM and SF with more skin additions, resembling cluster 3 in Chardonnay (Figure 4D). Among them, ethyl hexanoate and ethyl butyrate had an OAV >1 in control and all maceration treatments, suggesting that they are likely to contribute to the banana, green apple and strawberry notes of Italian Riesling wines (Table S3) [18]. All three terpenoids detected in Italian Riesling wines were grouped into cluster 1, where citronellol was the most abundant, followed by linalool and geraniol (Figure 4D). Terpenoids, a class of secondary phytoconstituents biosynthesized from acetyl coenzyme A, significantly contribute to wine’s characteristic aroma due to their low OTs [5]. The content of linalool was higher than the OT in all wines, reaching a maximum under 100% SF. Geraniol and citronellol exhibited OAVs >1 in all SF treatments and specifically in the 100% SF treatment, respectively (Table S3). Consequently, 100% SF treatment likely imparted a stronger floral and fruity aroma to the wines compared with other treatments. These findings may arise from the release of glycosidically bound terpenoid precursors from grape skins during maceration and their subsequent hydrolysis during fermentation [14,15,39], leading to higher terpenoid concentrations in skin-contact wines, especially in 100% SF treatment. The volatile compounds in cluster 2, predominantly esters and alcohols, were significantly elevated by SF treatments, whereas CM had a limited effect (Figure 4D). The OAVs of ethyl octanoate and isoamyl acetate were greater than 1 in all maceration treatments, especially in the SF treatments, indicating that the two esters may contribute more to the wine aroma (Table S3). In cluster 3, CM enhanced the contents of four volatile compounds, ethyl decanoate, decanoic acid, 4-methoxy-2,5-dimethylbenzaldehyde and 2,3-butanediol, which were consistently reduced by SF (Figure 4D). The reduction observed in SF treatment was likely due to phenolic compounds including flavonols and hydroxycinnamic acids derived from grape skins. These substances may alter yeast metabolism and inhibit relevant enzymes involved in the biosynthesis of these volatile compounds during fermentation [14,40].

**Figure 5:**
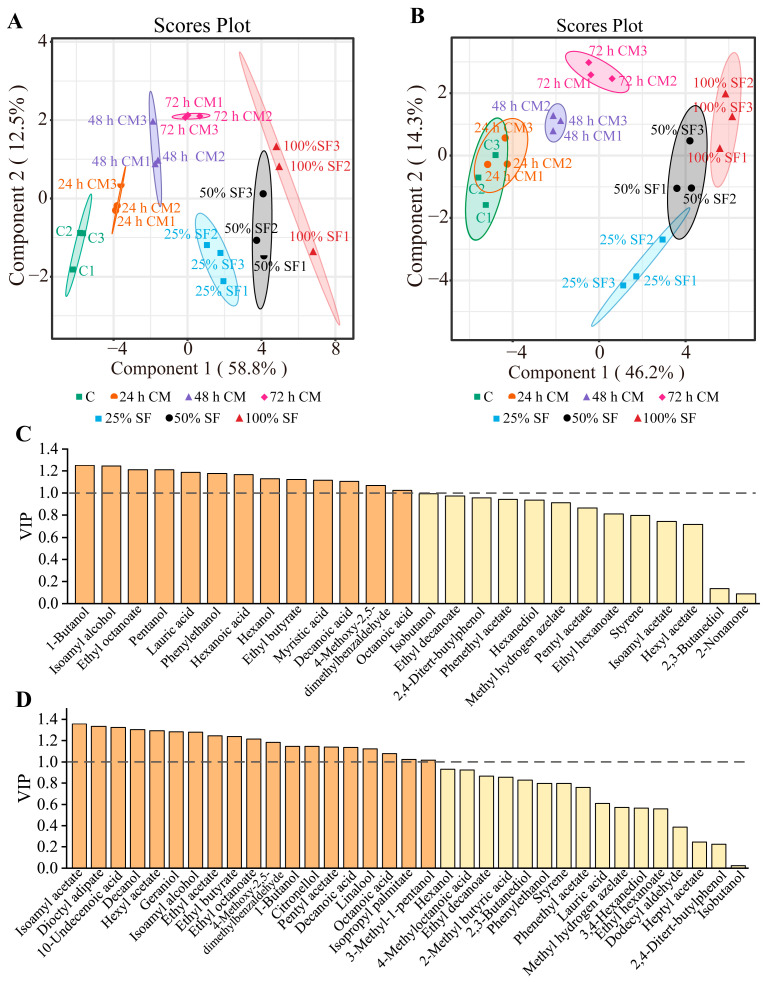
Orthogonal partial least squares discriminant analysis (OPLS-DA) (**A**,**B**) and VIP (**C**,**D**) of the volatile aroma compounds in Chardonnay (**A**,**C**) and Italian Riesling (**B**,**D**) white wines with different maceration processes. C, control. CM, cold maceration before fermentation. SF, skin-contact fermentation.

We then used orthogonal partial least squares discriminant analysis (OPLS-DA) to elucidate how different maceration treatments shape distinct wine aroma profiles. The OPLS-DA model demonstrated distinct separations, both between the control and various maceration treatments, as well as among the treatment groups themselves (Figure 5A), reflecting significant disparities in their volatile aroma compositions. For Italian Riesling, the model revealed clear separation between the control, 48 h CM, 72 h CM, and SF treatments, but there was an overlap between the control and the 24 h CM treatment (Figure 5B), suggesting no significant difference in aroma profile between the 24 h CM treatment and the control group. In addition, three CM treatments clustered around the control, and three SF treatments grouped together but were more distinctly separated from the control in both Chardonnay and Italian Riesling (Figure 5A,B). This indicated that the two maceration types differentially altered the aroma profiles, with SF treatments causing a greater deviation from the control than CM treatments; however, this statistical separation did not necessarily imply superiority in sensory quality. The R^2^ Y and Q^2^ values of the model validation permutation test were 0.985 (*p* < 0.005) and 0.948 (*p* < 0.005) for Chardonnay, and 0.98 (*p* < 0.005) and 0.955 (*p* < 0.005) for Italian Riesling, respectively (Figure S1A,B). The results of 200 random permutations confirmed that the two models showed good interpretability and predictive ability (Figure S1A,B). Moreover, thirteen and nineteen aroma substances with high VIP (>1) scores were identified as potential key differential compounds for distinguishing different treatments of Chardonnay and Italian Riesling, respectively (Figures S1C,D and Figure 5D). In Chardonnay, the aroma distinction between CM and SF treatments was mainly driven by higher alcohols and fatty acids in cluster 2 of the heatmap. These compounds are typically associated with alcoholic, chemical or fatty aromas, and can exert strong, even unpleasant off-flavors at high concentrations [12,13]. In contrast, Italian Riesling was mainly differentiated by esters and terpenoids, such as isoamyl acetate and hexyl acetate, which are secondary metabolites derived from fatty acid metabolism by yeast [6,38]. These esters with high OAVs in wine may contribute fruity aroma including banana, apple, pear notes, likely enhancing the perceived varietal typicality of macerating treated wines [5]. However, the complex interactions involving synergy and masking effects among volatile compounds, alongside interactions between volatiles and wine matrix components including ethanol, glycerol and caffeic acid may affect perceived aroma intensity in final wine [41,42]. Accordingly, the sensory evaluation was performed in the following section.

### 2.5. Color Analysis and Sensory Evaluation of Wines

#### 2.5.1. Color Analysis

In Chardonnay wines, notable increases in a* and b* values were observed under CM and SF treatments, with a* values increasing from −1.05 to −0.32 for 24 h CM and 25% SF treatments, and b* values increasing from 2.44 to 4.09–6.34, suggesting that both techniques can effectively reduce the green hue and enhance the yellow tone of wines (Table 2). The possible reason is the oxidation of phenolics during maceration, which generates *o*-quinone and yellow pigments, imparting a golden yellow hue to wines, as Carbone and Fiordiponti reported [43]. In addition, C* values were significantly enhanced in all maceration treatments except for 24 h CM, indicating that the maceration processes were effective in increasing the color saturation of Chardonnay wines (Table 2). As the proportion of skins increased in SF treatments, the a* values showed a tendency to first increase and then decrease. This phenomenon may be related to the release and oxidation of phenolics under low skin addition levels, whereas high skin addition may promote the secondary oxidative processes of phenolics or enhance the extraction of chlorophyll and other pigments from grape skins [43,44].

For Italian Riesling, 72 h CM, as well as 50% and 100% SF treatments, significantly decreased the L* values, which implied that prolonged CM and SF with high skin additions negatively affected the brightness of the wines. The 100% SF treatment significantly increased the a* values, which could help reduce the green hue of the wine. Moreover, SF treatments led to an increase in b* and C* values, while CM treatments resulted in a decrease in both (Table 2). It is possible that some yellow-hue compounds are absorbed by the grape solids or form precipitates with substances from the skins during CM, potentially resulting in a reduction in the yellow hue and color saturation of the wines [8].

Furthermore, the negative a* values were observed in all wine samples, reflecting a mild greenish tone associated with Chardonnay and Italian Riesling wines. However, Chardonnay and Italian Riesling white wines showed different CIELab parameters, particularly in b* value, with Italian Riesling showing higher yellow hue (Table 2). Moreover, the maceration processes differentially affected the color of the two wines. These differences were likely related to the intrinsic variations such as phenolic composition, phenolic content and cell wall structure between the two grape cultivars [45]. Nevertheless, the elevated b* values in Chardonnay and reduced L* values observed in Italian Riesling wines following prolonged CM and SF with more skin additions during fermentation may indicate excessive phenolic extraction and oxidative browning, leading to undesirable bitterness and a darker appearance.

#### 2.5.2. Sensory Evaluation

**Figure 6:**
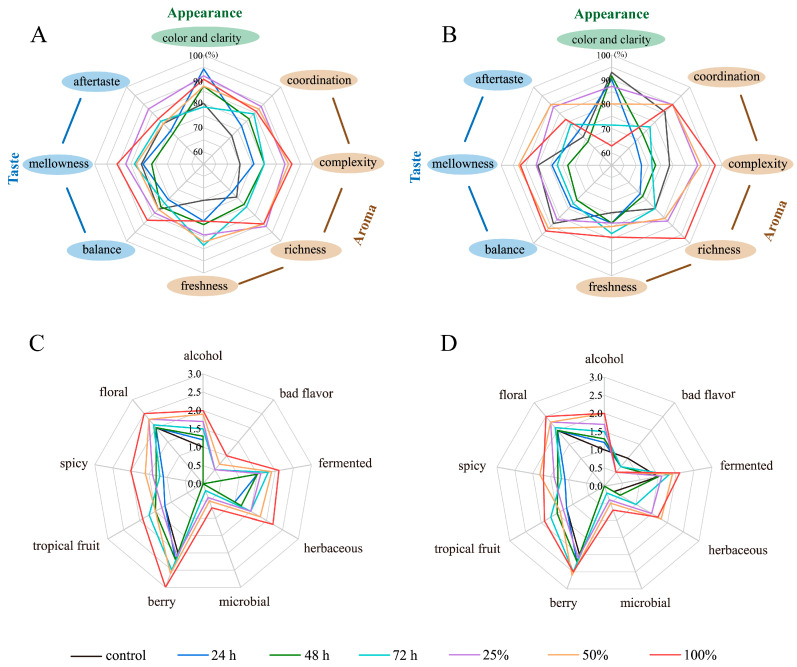
Sensory evaluation of Chardonnay and Italian Riesling white wines with different maceration processes. (**A**,**B**) Comprehensive sensory evaluation of Chardonnay (**A**) and Italian Riesling (**B**) white wines with different maceration processes. (**C**,**D**) Aroma profiles of Chardonnay (**C**) and Italian Riesling (**D**) white wines with different maceration processes.

Sensory evaluation plays an essential role in determining wine quality. Comprehensive sensory evaluation revealed that maceration treatments significantly influenced wine sensory characteristics. In both Chardonnay and Italian Riesling, SF treatments received higher scores for nose richness, nose complexity and palate mellowness (Figure 6A,B), likely reflecting enhanced phenolic extraction and volatile compound formation during fermentation with grape solids. Conversely, CM treatments preserved superior performance in aromatic freshness and balance mouthfeel, with wines exhibiting a more pronounced aftertaste. These sensory characteristics suggested CM treatments better preserved primary varietal aroma compounds, reduced oxidative impact and promoted the integration of perceived acidity, bitterness and wine body in oral perception. However, maceration treatments, particularly the 100% SF, elevated tannin and phenolic compound concentrations (Table 1), which may contribute to perceived bitterness and astringency on the palate, especially evident in aftertaste scores. These potentially negative mouthfeel attributes appear to be balanced by concomitant increases in viscosity and reductions in acidity, which may enhance perceived complexity and structure [11,12]. Indeed, previous research indicated that higher pH and phenolic content are positively associated with increased perceived viscosity and body [46], which may account for the high mellowness score observed in SF treatments.

The aroma profiles of Chardonnay and Italian Riesling revealed the distinct responses to variety and maceration treatment (Figure 6C,D). SF treatments significantly enhanced berry, tropical fruit and floral aromas in both varieties, especially at 100% SF, which was attributed to their higher ester levels. Ethyl octanoate, ethyl hexanoate, isoamyl acetate and ethyl butyrate conferred fresh grape, pear, strawberry and banana-like aromas with their values exceeding perception thresholds (Figure 5A and Table S2) [7,21]. However, the increases in undesirable odors observed in SF treatments should be noted, which were potentially due to the accumulation of fatty acids produced by yeast metabolism [5,47]. In Italian Riesling wines, the maceration treatments resulted in more pronounced herbaceous character perceived as tea-like (Figure 6D), which may be linked to an increased concentration of higher alcohol and terpenes [13]. Both varieties exhibited no significant changes in alcohol or spicy aromas, indicating that these treatments primarily affected specific volatile aroma compounds such as esters, acids and terpenes rather than ethanol and higher alcohols.

Overall, the effectiveness of CM and SF techniques were highly dependent on wine grape variety, highlighting the critical importance of customizing winemaking protocols. Moreover, SF treatment is more suitable for white wines intended to emphasize complexity and texture, whereas CM treatment is better suited for fresh and aromatic white wines. This allows winemakers to select processing techniques to craft distinct white wine, catering to diverse consumer preferences.

## 3. Materials and Methods

### 3.1. Grapes and Winemaking Procedures

Fresh Chardonnay and Italian Riesling grapes (total soluble solids: 18.43 ± 0.12 ^°^Brix and 18.07 ± 0.06 ^°^Brix, respectively), harvested in 2022 vintage by Yantai Cascade Valley Winery in Yantai, China, were de-stemmed and crushed. Chardonnay must had 184.26 ± 1.57 g/L total sugar, 8.40 ± 0.15 g/L total acid and pH of 3.01 ± 0.01. Italian Riesling must had 178.34 ± 3.02 g/L total sugar, 7.50 ± 0.25 g/L total acid and pH of 3.15 ± 0.02. Potassium metabisulfite (60 mg/L, Enartis, San Martino Trecate, Italy) and pectinase (30 mg/L, Laffort, Bordeaux, France) were added to grape must and stirred uniformly. The winemaking experiments were conducted at a laboratory scale, with 4 kg of grape must used in each fermentation. Grape must was subjected to different processing procedures in different treatments, and all treatments were performed in triplicate. In the control group, wines were prepared using the traditional white winemaking process. Grape must was pressed in 5 L glass flasks and clarified at 4 °C for 24 h. In CM treatments, grape must containing solids was incubated at 4 ± 1 °C for 24, 48 and 72 h (designated as 24 h CM, 48 h CM and 72 h CM treatments) and then pressed. The juice in the control group and CM treatments was rewarmed to 20 °C, and then inoculated with 150 mg/L *Saccharomyces cerevisiae* EC1118 (Lallemand, Montreal, QC, Canada) to start the fermentation. In SF treatments, 25, 50 and 100% of the 4 kg of grape must was thoroughly mixed with juice pressed from the remaining 75, 50, and 0% must, corresponding to the 25% SF, 50% SF and 100% SF groups, respectively. *S. cerevisiae* EC1118 was subsequently inoculated for alcohol fermentation, and the grapes were pressed at the end of the fermentation. All treatments were performed in triplicate. Fermentations were conducted at 20 ± 1 °C, and the ^°^Brix value were monitored daily. After the fermentation finished (when ^°^Brix value stopped decreasing), 100 mg/L potassium metabisulfite was added. Finally, the wines were centrifuged at 3000 rpm for 5 min to remove the foot, then bottled and stored at 4 °C for further analysis.

### 3.2. Basic Physicochemical Parameter Analysis

The residual sugar, alcohol, total acid, volatile acid and pH in the wine samples were detected by OenoFoss wine auto analyzer (FOSS, Hilleroed, Denmark). Total tannins were determined with reference to the method of Girard et al. with tannic acid as the standard [48]. Total phenol were determined according to the method of Lan et al. with gallic acid (Alta, Tianjin, China) as the standard [49].

### 3.3. Quantitative Analysis of Phenolics

Phenolics in wine samples were determined by high performance liquid chromatography (HPLC) using a Waters xBridge^®^C18 column (4.6 mm × 250 mm, 5 μm) (Waters, Milford, MA, USA). The analyses for different phenolic compounds were performed as follows, with a flow rate of 1.0 mL/min and detection by DAD. Phenolic acids were analyzed following the method described by Di Lecce et al. with modifications [34]. The mobile phase consisted of A (methanol:acetic acid:water = 10:2:88, *v*/*v*) and B (methanol), with the following gradient program: 0–25 min, 0–15% B; 25–45 min, 15–50% B; 45–53 min, 50–0% B. The column temperature was set at 30 °C, and the detection wavelength was 280 nm, except for gentisic acid which was detected at 320 nm [50]. Flavane-3-ols were determined according to a previously published method by Yao et al. [13]. Mobile phase A was water (0.2% formic acid, *v*/*v*), and mobile phase B was methanol. The elution procedure was 96–75% A for 0–11 min, 75–65% A for 11–40 min, 65–0% A for 40–50 min, 0% A for 50–60 min and 96% A for 60–65 min. The column temperature was 30 °C, and the detection wavelength was 360 nm. Flavonols were analyzed following the method of Paladines-Quezada et al. [51]. Mobile phase A was ethylamine/methanol/water/tetrahydrofuran (19:5:76:1, *v*/*v*), and mobile phase B was ethylamine/methanol/water (55:15:30, *v*/*v*). The gradient program was as follows: 0–30 min, 95–70% A; 30–44 min, 70–65% A; 44–52 min, 65–5% A; 52–60 min, 5–95% A. The column temperature was 40 °C, and the detection wavelength was 360 nm. Phenolics were identified by comparison with standard retention times and spectral characteristics. Quantification was performed using the external standard method. Method validation was conducted, and the detailed parameters are summarized in Table S4.

### 3.4. Identification and Quantification of Volatile Compounds by HS-SPME-GC-MS

Volatile compounds were analyzed by headspace solid-phase microextraction-gas HS-SPME-GC-MS according to a previous study with slight modifications [52]. Briefly, wine samples (3 mL) with 2-octanol (Macklin, Shanghai, China) as an internal standard (2 µL, 0.822 g/L), NaCl (0.5 g, Macklin, Shanghai, China) and a magnetic rotor were placed in a 10 mL headspace vial. Volatile compounds were extracted at 40 °C, 500 r/min for 50 min using a DVB/CAR/PDMS (50/30 μm) extraction fiber (Supelco, Bellefonte, PA, USA). The fiber was then inserted into the GC injection port, and volatile compounds were desorbed at 250 °C for 8 min. GC-MS analysis were conducted using a gas chromatograph TSQ8000evo (ThermoFisher Inc., Waltham, MA, USA), equipped with the SUPELCOWAX10 capillary column (60 m × 0.25 mm, 0.25 μm, Supelco, Bellefonte, PA, USA). High-purity helium was used as the mobile phase at a flow rate of 1.2 mL/min. The injector temperature was set at 250 °C. The GC procedure was as follows: initial temperature of 50 °C held for 1 min, increased to 180 °C at a rate of 3 °C/min, then increased to 230 °C at a rate of 20 °C/min and held for 15 min. The MS was operated in the electron ionization (EI) mode, and the scanning range was 40 to 450 *m*/*z*. The interface and ion source temperatures were set at 280 °C and 230 °C, respectively.

The volatile compounds were identified by matching each aroma component with the NIST 17.0 standard library. The volatile compounds were quantified by multiplying the peak area ratio of each volatile compound to the internal standard by the internal standard concentration.

### 3.5. Color Measurement

The wine samples were filtered through 0.45 μm aqueous filter membranes and determined using a ultraviolet-visible spectrophotometer (Agilent Technologies Inc., Santa Clara, CA, USA) following the method of Fan et al. [53]. The absorbance of the wine samples at 450, 520, 570, and 630 nm was determined using pure water as a blank control, and CIELab parameters including L* (lightness), a* (green/red), b* (blue/yellow) and C* (saturation) were calculated. The results were repeated three times for each sample.

### 3.6. Sensory Analysis

Wine sensory profiles were evaluated using a quantitative analysis method, based on the method of Wang et al. with slight modifications [54]. Seven panelists (four males and three females, aged 23 to 45) from Cascade Valley Winery and Binzhou Medical University voluntarily participated in the sensory evaluation. All participants possessed relevant educational or professional backgrounds and received four training sessions within one month using physical reference standards to align scoring criteria for specific wine attributes. Prior to the formal evaluation, all panelists were assessed for consistency and accuracy through a test of wine appearance, aroma and taste. The formal trial only proceeded once the identification accuracy for all sensory terms exceeded 95% for all panelists. Panelists worked independently in individual booths compliant with ISO 8589 standards [55]. Wine samples were presented in clear, standardized glasses labeled with three-digit random codes and served in a randomized order. Comprehensive sensory evaluation contained appearance, aroma and taste, with the scoring criteria as follows: appearance (color and clarity, 10 points), aroma (coordination, 10 points; complexity, 10 points; richness, 10 points; freshness, 10 points), and taste (balance, 20 points; mellowness, 20 points; aftertaste, 10 points). Radar charts were constructed based on the percentage of each score relative to the full score. Nine aroma attributes including alcohol odor, floral, spicy, tropical fruit, berry, microbial, herbaceous, fermented and bad flavor were assessed on a three-point scale by smelling, with higher scores corresponding to stronger intensity. All wine samples were evaluated in triplicate by each panelist, and final scores represented the mean value of all replicates and panelists.

### 3.7. Data Analysis

The results were presented as mean ± standard deviation. One-way analysis of variance (ANOVA) was performed by SPSS 17.0 software (SPSS Inc., Chicago, IL, USA). Origin 2018 software (OriginLab Corporation, Northampton, MA, USA) was used to plot the °Brix values and flavor radar charts for the wine samples with different maceration processes. PCA was performed using SIMCA 14.1 software (Umetrics, Umea, Sweden). Heat maps, cluster analysis and OPLS-DA were performed using Metware Cloud, a free online platform for data analysis (https://cloud.metware.cn).

## 4. Conclusions

This study revealed that CM and SF with different maceration times and skin addition ratios significantly affected the chemical and sensory profiles of Chardonnay and Italian Riesling white wines in Yantai wine region. The effects are highly dependent on maceration treatments and grape variety. Both CM and SF treatments substantially enhanced the extraction of phenolics and promoted the formation of key aromatic metabolites. However, excessive maceration also led to undesirable color and off-odors. Winemakers should optimize maceration parameters to avoid these negative effects. These findings may provide insights for the optimization of region-specific maceration in white wine production. Further research should focus on elucidating the dynamics of phenolics and volatile compounds during the maceration process, which will assist in optimizing the winemaking process while reducing quality defects.

## Figures and Tables

**Table 1:** Basic physicochemical indexes of Chardonnay and Italian Riesling white wines with different maceration processes.

	Control	Cold Maceration			Skin-Contact Fermentation		
		24 h	48 h	72 h	25%	50%	100%
Chardonnay							
Residual sugar (g/L)	2.53 ± 0.45 ^c^	2.20 ± 0.17 ^c^	2.80 ± 0.70 ^c^	2.86 ± 0.35 ^bc^	3.60 ± 0.26 ^ab^	3.70 ± 0.26 ^a^	3.60 ± 0.20 ^a^
Total acid (g/L)	8.50 ± 0.15 ^a^	7.24 ± 0.22 ^c^	7.95 ± 0.39 ^b^	7.87 ± 0.30 ^b^	7.25 ± 0.19 ^c^	7.57 ± 0.27 ^bc^	7.80 ± 0.13 ^b^
pH	2.99 ± 0.03 ^d^	3.15 ± 0.03 ^bc^	3.18 ± 0.02 ^b^	3.24 ± 0.02 ^a^	3.15 ± 0.02 ^bc^	3.12 ± 0.02 ^c^	3.18 ± 0.02 ^b^
Volatile acid (g/L)	0.57 ± 0.05 ^b^	0.44 ± 0.03 ^c^	0.53 ± 0.02 ^bc^	0.79 ± 0.08 ^a^	0.42 ± 0.08 ^c^	0.46 ± 0.03 ^bc^	0.42 ± 0.06 ^c^
Alcohol (%*v*/*v*)	10.20 ± 0.20 ^a^	10.03 ± 0.15 ^a^	9.93 ± 0.15 ^a^	9.83 ± 0.21 ^a^	9.86 ± 0.05 ^a^	9.85 ± 0.26 ^a^	9.93 ± 0.20 ^a^
Total tannin (mg/L)	123.68 ± 4.15 ^d^	124.59 ± 8.93 ^d^	153.12 ± 4.31 ^c^	205.56 ± 5.82 ^b^	146.81 ± 3.72 ^c^	152.59 ± 4.17 ^c^	226.66 ± 1.83 ^a^
Total phenol (mg/L)	205.29 ± 8.19 ^c^	210.88 ± 1.88 ^c^	213.08 ± 10.44 ^bc^	232.55 ± 2.34 ^b^	218.52 ± 2.95 ^bc^	288.25 ± 7.05 ^a^	290.04 ± 21.75 ^a^
Italian Riesling							
Residual sugar (g/L)	2.60 ± 0.50 ^c^	2.66 ± 0.11 ^c^	2.83 ± 0.05 ^bc^	2.63 ± 0.35 ^c^	3.50 ± 0.26 ^b^	3.63 ± 0.25 ^a^	3.30 ± 0.26 ^ab^
Total acid (g/L)	7.30 ± 0.20 ^b^	7.30 ± 0.17 ^b^	7.43 ± 0.12 ^b^	8.00 ± 0.17 ^a^	6.76 ± 0.15 ^c^	6.67 ± 0.15 ^c^	6.30 ± 0.17 ^d^
pH	3.11 ± 0.022 ^f^	3.18 ± 0.03 ^e^	3.25 ± 0.01 ^bc^	3.28 ± 0.01 ^b^	3.20 ± 0.02 ^de^	3.22 ± 0.03 ^cd^	3.33 ± 0.02 ^a^
Volatile acid (g/L)	0.71 ± 0.09 ^a^	0.68 ± 0.03 ^a^	0.56 ± 0.03 ^b^	0.68 ± 0.05 ^a^	0.38 ± 0.07 ^c^	0.47 ± 0.03 ^bc^	0.50 ± 0.05 ^b^
Alcohol (%*v*/*v*)	9.60 ± 0.26 ^a^	9.43 ± 0.42 ^a^	9.83 ± 0.25 ^a^	9.53 ± 0.25 ^a^	9.70 ± 0.55 ^a^	9.40 ± 0.30 ^a^	9.73 ± 0.15 ^a^
Total tannin (mg/L)	139.41 ± 6.61 ^f^	148.69 ± 1.97 ^e^	166.02 ± 2.41 ^d^	195.99 ± 3.87 ^b^	148.34 ± 2.68 ^e^	181.99 ± 3.59 ^c^	225.28 ± 3.78 ^a^
Total phenol (mg/L)	198.15 ± 5.68 ^d^	198.67 ± 7.46 ^d^	196.18 ± 8.19 ^d^	217.81 ± 6.60 ^bc^	214.92 ± 1.75 ^c^	229.39 ± 3.91 ^b^	247.87 ± 11.47 ^a^

The concentration values are from triplicate experiments (mean ± standard deviation [SD]). Significance analysis was performed only between treatments for the same indicator. Different letters (^a–f^) in the same row indicate statistically significant differences according to Waller–Duncan’s test (*p* < 0.05).

**Table 2:** CIELab color parameters of Chardonnay and Italian Riesling white wine with different maceration processes.

Parameters	Control	Cold Maceration			Skin-Contact Fermentation		
		24 h	48 h	72 h	25%	50%	100%
Chardonnay							
L*	96.65 ± 0.67 ^a^	98.82 ± 0.11 ^a^	97.89 ± 0.79 ^a^	96.43 ± 2.76 ^a^	98.05 ± 0.06 ^a^	97.88 ± 0.11 ^a^	97.98 ± 0.39 ^a^
a*	−1.05 ± 0.68 ^a^	−0.32 ± 0.17 ^b^	−0.45 ± 0.11 ^b^	−0.45 ± 0.11 ^b^	−0.32 ± 0.11 ^b^	−0.52 ± 0.04 ^ab^	−0.78 ± 0.13 ^ab^
b*	2.44 ± 0.51 ^d^	4.09 ± 0.16 ^c^	4.71 ± 1.41 ^bc^	6.34 ± 0.72 ^a^	5.42 ± 0.13 ^ab^	5.49 ± 0.16 ^ab^	6.26 ± 0.32 ^a^
C*_ab_	3.80 ± 0.32 ^c^	4.12 ± 0.16 ^c^	6.05 ± 0.17 ^ab^	6.36 ± 0.72 ^a^	5.42 ± 0.12 ^b^	5.52 ± 0.16 ^b^	6.32 ± 0.34 ^a^
Italian Riesling							
L*	97.78 ± 0.32 ^a^	99.11 ± 0.09 ^a^	98.74 ± 0.33 ^a^	95.28 ± 0.46 ^bc^	97.37 ± 0.09 ^ab^	95.10 ± 2.68 ^c^	87.46 ± 1.21 ^d^
a*	−0.83 ± 0.25 ^ab^	−0.69 ± 0.09 ^ab^	−0.66 ± 0.05 ^ab^	−0.64 ± 0.11 ^ab^	−0.98 ± 0.17 ^ab^	−1.15 ± 0.11 ^a^	−0.52 ± 0.58 ^b^
b*	7.06 ± 1.20 ^b^	3.52 ± 0.08 ^d^	4.31 ± 0.52 ^cd^	5.44 ± 0.03 ^c^	8.25 ± 0.51 ^ab^	9.10 ± 0.97 ^a^	8.55 ± 1.08 ^ab^
C*_ab_	7.11 ± 0.22 ^b^	3.59 ± 0.07 ^d^	4.36 ± 0.52 ^d^	5.47 ± 0.03 ^c^	8.31 ± 0.49 ^a^	9.17 ± 0.97 ^a^	8.58 ± 0.06 ^a^

CIELab color parameters are from triplicate experiments (mean ± standard deviation [SD]). Significance analysis was performed only between treatments for the same indicator. Different letters (^a–d^) in the same row indicate statistically significant differences according to Waller–Duncan’s tests (*p* < 0.05).

## Data Availability

The original contributions presented in this study are included in the article/Supplementary Materials. Further inquiries can be directed to the corresponding authors.

## Supplementary Materials

The following supporting information can be downloaded at: https://www.mdpi.com/article/10.3390/molecules31132223/s1, Table S1: Phenolic content of Chardonnay and Italian Riesling white wines with different maceration processes; Table S2: Volatile aroma compounds in Chardonnay white wine with different maceration processes; Table S3: Volatile aroma compounds in Italian Riesling white wine with different maceration processes; Table S4: Recovery, repeatability and LOD of phenolic content by HPLC; Figure S1: Model validation permutation test (A–B) and S-plot (C–D) of the volatile aroma compounds in Chardonnay (A and C) and Italian Riesling (B and D) white wines with different maceration processes. References [56,57,58] are cited in the Supplementary.
