# Supplementary material for: Maceration in White Winemaking: Enhancing Phenolics, Volatile Aromas and Sensory Characteristics of Chardonnay and Italian Riesling Wines
Source: Molecules. 2026 Jun 24;31(13):2223. doi: 10.3390/molecules31132223 (PMC13363409; doi:10.3390/molecules31132223)
Supplement: Supplementary file 1 [file molecules-31-02223-s001.zip › molecules-4342545-supplementary.pdf]

Table S1. Phenolic content of Chardonnay and Italian Riesling white wines with different maceration processes.

	Content (mg/L)	Control	Cold maceration			Skin-contact fermentation		
			24 h	48 h	72 h	25%	50%	100%
Chardonnay	**Phenolic acids**	**9.13**	**9.6**	**11.23**	**14.51**	**10.01**	**11.79**	**13.19**
	Gallic acid	0.54±0.06[e]	0.82±0.17[de]	1.30±0.08[d]	2.25±0.22[c]	2.66±0.02[c]	4.71±0.33[b]	6.29±0.52[a]
	Protocatechuic acid	2.52±0.56[abc]	2.53±0.49[abc]	2.88±0.19[ab]	3.04±0.73[a]	1.99±0.27[bc]	1.81±0.23[c]	1.58±0.74[c]
	Gentisic acid	0.28±0.05[e]	0.39±0.01[d]	0.51±0.04[bc]	0.64±0.06[a]	0.43±0.04[cd]	0.54±0.05[b]	0.47±0.04[bcd]
	Chlorogenic acid	1.93±0.37[a]	2.21±0.56[a]	2.34±0.31[a]	2.36±0.18[a]	1.28±0.10[b]	1.23±0.22[b]	0.90±0.25[b]
	Caffeic acid	1.86±0.01[b]	1.59±0.09[b]	1.79±0.50[b]	3.52±0.24[a]	1.59±0.25[b]	1.56±0.16[b]	2.05±0.53[b]
	p-Coumaric acid	0.97±0.03[c]	1.10±0.04[bc]	1.41±0.4[ab]	1.62±0.06[a]	1.05±0.12[bc]	1.05±0.08[c]	1.11±0.20[bc]
	Ferulic acid	1.03±0.21[a]	0.96±0.03[a]	1.00±0.08[a]	1.08±0.31[a]	1.01±0.36[a]	0.89±0.14[a]	0.79±0.20[a]
	**Flavane-3-ols**	**24.77**	**29.04**	**31.46**	**31.25**	**26.19**	**32.38**	**31.67**
	C	0.42±0.15[c]	0.35±0.11[c]	0.57±0.04[bc]	0.70±0.05[b]	0.76±0.08[b]	1.27±0.15[a]	1.50±0.16[a]
	EC	0.62±0.13[b]	0.61±0.04[b]	0.63±0.06[b]	0.70±0.06[b]	0.63±0.11[b]	0.68±0.02[b]	0.93±0.02[a]
	EgC	23.07±4.27[c]	27.37±0.88[c]	29.10±1.18[a]	28.69±1.12[a]	23.64±1.55[bc]	28.96±0.83[a]	27.67±1.97[ab]
	EgCg	0.66±0.27[c]	0.71±0.36[c]	1.16±0.02[b]	1.16±0.11[b]	1.16±0.01[a]	1.47±0.14[ab]	1.57±0.09[a]
	**Flavonol**	**3.34**	**3.80**	**4.07**	**5.59**	**4.21**	**6.65**	**6.73**
	Rutin	1.93±0.06[b]	1.69±0.08[c]	1.85±0.17[bc]	2.74±0.01[a]	1.50±0.12[d]	1.86±0.06[bc]	1.96±0.02[b]
	Quercitrin	0.41±0.06[d]	1.12±0.13[c]	1.27±0.02[c]	1.67±0.05[b]	1.80±0.05[b]	3.95±0.19[a]	4.11±0.41[a]
	Myricetin	0.58±0.15[ab]	0.66±0.01[a]	0.58±0.03[ab]	0.68±0.01[a]	0.63±0.01[ab]	0.54±0.01[b]	0.33±0.01[c]
	Quercetin	0.22±0.01[b]	0.15±0.01[c]	0.17±0.02[c]	0.32±0.02[a]	0.11±0.01[d]	0.11±0.01[d]	0.14±0.03[cd]

	Galangin	0.20±0.02^{a}	0.18±0.03^{a}	0.20±0.01^{a}	0.18±0.02^{a}	0.17±0.02^{a}	0.19±0.07^{a}	0.19±0.02^{a}
**Italian Riesling**	**Phenolic acids**	**11.12**	**10.36**	**11.03**	**11.07**	**13.49**	**14.48**	**15.88**
	Gallic acid	1.33±0.16^{f}	1.42±0.09^{f}	1.45±0.06^{e}	1.91±0.07^{d}	3.27±0.30^{c}	4.79±0.19^{b}	6.44±0.34^{a}
	Protocatechuic acid	4.41±0.69^{a}	4.44±0.93^{a}	4.28±0.33^{a}	4.30±0.89^{a}	4.67±1.15^{a}	4.58±1.77^{a}	4.23±1.95^{a}
	Gentisic acid	0.52±0.06^{b}	0.43±0.02^{c}	0.36±0.01^{d}	0.30±0.05^{d}	0.56±0.03^{b}	0.67±0.02^{a}	0.69±0.04^{a}
	Chlorogenic acid	0.88±0.07^{ab}	0.83±0.04^{b}	0.85±0.03^{ab}	0.87±0.09^{ab}	0.82±0.01^{b}	0.93±0.04^{a}	0.85±0.02^{ab}
	Caffeic acid	2.79±0.48^{a}	2.03±1.48^{a}	2.74±0.94^{a}	2.30±1.13^{a}	2.85±0.59^{a}	2.05±0.97^{a}	2.14±0.44^{a}
	p-Coumaric acid	0.22±0.02^{d}	0.28±0.04^{cd}	0.34±0.01^{bc}	0.35±0.08^{bc}	0.32±0.02^{c}	0.42±0.06^{ab}	0.48±0.03^{a}
	Ferulic acid	0.97±0.14^{a}	0.93±0.09^{a}	1.01±0.08^{a}	1.04±0. 15^{a}	1.00±0.06^{a}	1.04±0.05^{a}	1.05±0.06^{a}
	**Flavane-3-ols**	**26.09**	**25.98**	**32.14**	**39.29**	**30.51**	**36.62**	**42.39**
	C	0.20±0.01^{d}	0.20±0.02^{d}	0.75±0.18^{bc}	0.94±0.03^{b}	0.65±0.19^{c}	0.76±0.07^{bc}	1.81±0.13^{a}
	EC	1.35±0.34^{a}	1.43±0.30^{a}	1.37±0.04^{a}	1.69±0.29^{a}	1.37±0.18^{a}	1.35±0.07^{a}	1.36±0. 19^{a}
	EgC	23.25±2.05^{cd}	22.98±2.10^{d}	28.76±2.75^{b}	35.16±2.36^{a}	26.88±1.28^{bc}	32.88±0.48^{a}	36.59±1.02^{a}
	EgCg	1.29±0.08^{cd}	1.28±0.04^{cd}	1.26±0.09^{d}	1.50±0.02^{bc}	1.61±0.11^{b}	1.63±0.02^{b}	2.63±0.23^{a}
	**Flavonol**	**5.39**	**7.38**	**7.89**	**8.65**	**7.10**	**7.99**	**11.5**
	Rutin	3.19±0.40^{c}	3.29±0.61^{c}	3.26±0.08^{c}	4.23±0.83^{b}	3.51±0.04^{bc}	4.04±0.24^{bc}	6.50±0.04^{a}
	Quercitrin	0.39±0.01^{e}	2.29±0.20^{d}	3.26±0.08^{bc}	3.23±0.83^{bc}	2.52±0.15^{cd}	3.29±0.14^{b}	4.22±0.42^{a}
	Myricetin	0.87±0.11^{a}	0.69±0.11^{c}	0.92±0.05^{a}	0.71±0.08^{bc}	0.45±0.14^{d}	0.35±0.01^{d}	0.34±0.01^{d}
	Quercetin	0.94±0.11^{ab}	1.11±0.32^{a}	0.45±0.01^{c}	0.48±0.02^{c}	0.62±0.34^{bc}	0.31±0.02^{c}	0.44±0.12^{c}

Values with different letters in the same row are significantly different ($p < 0.05$).

Table S2. Volatile aroma compounds in Chardonnay white wine with different maceration processes.

Content (μg/L)	Control	Cold maceration			Skin-contact fermentation			Threshold (μg/L)	Description
		24 h	48 h	72 h	25%	50%	100%		
**Alcohol**	**1942.39**	**2366.83**	**2567.58**	**3166.85**	**3220.76**	**3750.9**	**4257.35**		
Isoamyl alcohol	1076.14±118.34^{e}	1370.77±211.67^{de}	1473.41±119.45^{d}	1835.47±207.34^{c}	2087.41±101.47^{bc}	2241.78±152.94^{b}	2567.22±129.01^{a}	30,000 [1]	Harsh, stale, fusel odour [1]
1-Butanol	27.31±1.34^{e}	29.92±1.11^{e}	47.20±2.78^{d}	65.47±6.37^{c}	54.71±4.33^{d}	89.67±5.39^{b}	107.43±6.11^{a}	15,000 [2]	Chemical, solvent, bitter [2]
1-Hexanol	247.14±31.39^{e}	274.39±27.30^{de}	341.24±57.23^{cd}	417.82±74.20^{bc}	348.75±15.71^{cd}	442.57±27.98^{ab}	514.28±58.31^{a}	1,100 [2]	Herbaceous, grass, woody [2]
Hexanediol	37.24±5.34^{b}	36.86±2.79^{b}	37.31±5.17^{b}	41.24±11.11^{b}	47.24±7. 19^{ab}	50.54±12.10^{ab}	62.71±15.43^{a}	-	-
Phenylethanol	459.73±22.47^{d}	553.04±10.94^{c}	562.27±23.47^{c}	682.26±31.74^{c}	576.14±38.89^{b}	758.01±57.11^{a}	802.20±38.39^{a}	14,000 [2]	Floral, rose [2]
Isobutanol	24.57±1.39^{c}	22.88±1.34^{c}	23.41±2.71^{c}	24.18±4.33^{c}	23.15±1.21^{c}	43.82±3.11^{b}	64.21±1.37^{a}	40,000 [2]	Medicinal, phenolic [2]
2,3-Butanediol	53.10±5.11^{a}	57.10±3.91^{a}	53.10±5.10^{a}	51.10±1.51^{a}	50.79±3.24^{a}	57.36±5.33^{a}	56.41±5.69^{a}	15,000 [1]	Fruity, fresh [1]
1-Pentanol	17.16±0.91^{e}	21.87±1.24^{de}	29.64±3.64^{d}	49.31±7.20^{c}	32.57±5.77^{d}	67.15±4.98^{b}	82.89±10.78^{a}	64,000 [2]	Balsamic, bitter almond [2]
**Acid**	**3523.93**	**3448.26**	**3885.99**	**4839.51**	**4307.78**	**6501.98**	**7148.67**		
Hexanoic acid	247.31±29.78^{d}	297.43±25.31^{cd}	307.12±11.30^{cd}	336.82±39.33^{c}	317.91±24.12^{c}	487.26±44.39^{b}	554.33±37.24^{a}	420 [1]	Sour, vinegar, cheese, sweaty, chemical [1]
Decanoic acid	1774.23±337.19^{b}	1763.21±218.49^{b}	1976.34±229.48^{b}	2347.31±358.64^{b}	2038.77±249.36^{b}	3324.67±559.11^{a}	3647.19±409.34^{a}	1,000 [1]	Vinegar, animal, fatty, rancid, citrus, phenolic [1]
Octanoic acid	1371.64±422.85^{c}	1247.98±527.33^{c}	1457.14±137.65^{c}	1974.31±336.47^{abc}	1786.47±255.31^{bc}	2471.15±449.37^{ab}	2676.99±371.45^{a}	500 [1]	Goat rancid cheese, fatty, oily, acetic [1]
Lauric acid	129.41±13.27^{d}	138.07±14.69^{d}	143.34±11.87^{d}	179.12±10.24^{c}	162.78±24.17^{cd}	216.29±12.87^{b}	266.25±23.71^{a}	1,000 [3]	Dried fruit, metallic taste [3]
Myristic acid	1.34±0.28^{c}	1.57±0. 11^{c}	2.05±0.07^{bc}	1.95±0. 13^{bc}	1.85±0.37^{c}	2.61±0.07^{b}	3.91±0.84^{a}	-	-

**Ester**	**3733.66**	**4117.47**	**5057.61**	**5854.43**	**4546.78**	**5304.45**	**6577.61**		
Pentyl acetate	1385.14±109.34^{c}	1439.27±297.36^{c}	1863.78±187.32^{bc}	2039.71±247.39^{ab}	1642.38±125.35^{bc}	1834.92±247.36^{bc}	2367.11±375.38^{a}	-	-
Isoamyl acetate	168.17±45.97^{c}	225.26±18.97^{bc}	279.34±24.64^{ab}	347.68±48.64^{a}	227.07±14.37^{bc}	278.81±22.39^{ab}	334.82±101.37^{a}	30 [4]	Fresh, sweet, fruity [4]
Hexyl acetate	293.63±24.69^{b}	323.10±34.97^{b}	320.13±87.45^{b}	437.70±64.35^{a}	318.32±54.36^{b}	378.14±45.67^{ab}	442.63±24.64^{a}	670 [4]	Green, fruity, sweet, fatty, fresh [4]
Phenethyl acetate	471.83±98.31^{d}	489.31±57.34^{d}	697.44±87.66^{bc}	734.96±105.64^{b}	527.12±69.34^{cd}	752.34±107.64^{b}	974.31±98.34^{a}	1,800 [5]	Fruity, rose [5]
Ethyl octanoate	223.99±56.34^{c}	236.14±29.64^{c}	297.21±18.34^{c}	332.01±54.81^{c}	493.46±106.47^{b}	549.18±54.39^{b}	695.70±104.67^{a}	5 [1]	Fruity, sweet, waxy [1]
Ethyl decanoate	391.71±29.37^{a}	416.60±65.34^{a}	437.79±89.34^{a}	457.42±78.36^{a}	256.59±55.31^{b}	210.76±54.37^{b}	217.23±57.34^{b}	200 [1]	Fruity, grape, pear, apple [1]
Ethyl hexanoate	751.98±102.34^{b}	894.02±106.78^{b}	1039.22±279.34^{ab}	1327.21±247.69^{a}	951.36±164.35^{ab}	1132.47±241.35^{ab}	1320.17±98.66^{a}	80 [2]	Banana, green apple [2]
Ethyl butyrate	47.21±9.67^{d}	69.46±21.97^{cd}	95.06±24.30^{bc}	124.48±11.64^{ab}	95.31±21.67^{bc}	121.74±14.87^{ab}	158.33±24.38^{a}	20 [6]	Sour fruit, fruity, strawberry [6]
Methyl hydrogen azelate	nd	24.31±1.39^{d}	27.64±1.36^{cd}	53.26±11.36^{ab}	35.17±14.69^{bcd}	46.09±6.55^{bc}	67.31±12.44^{a}	-	-
**Aldehyde and Ketone**	**18.79**	**17.25**	**19.93**	**17.99**	**18.41**	**20.44**	**21.7**		
4-Methoxy-2,5-dimethylbenzaldehyde	0.03±0.00^{d}	nd	1.62±0.24^{bc}	1.74±0.48^{bc}	0.57±0.00^{cd}	2.37±1.44^{b}	3.74±0.54^{a}	-	-
2-Nonanone	18.76±2.67^{a}	17.25±2.37^{a}	18.31±2.74^{a}	16.25±9.32^{a}	17.84±5.32^{a}	18.07±6.78^{a}	17.96±2.35^{a}	15 [3]	Grass, orange, lemon oil [3]
**Others**	**46.25**	**85.59**	**177.74**	**208.82**	**99.22**	**128.97**	**248.2**		
Styrene	45.21±14.37^{c}	70.72±19.74^{c}	156.70±24.82^{ab}	184.32±67.38^{a}	83.13±22.34^{c}	99.82±21.37^{bc}	217.31±48.80^{a}	250 [7]	Sweet [7]
2,4-Ditert-butylphenol	1.04±0.37^{c}	14.87±6.75^{b}	21.04±5.34^{ab}	24.50±4.31^{ab}	16.09±10.71^{b}	29. 15±2.37^{a}	30.89±1.71^{a}	200 [3]	Carbonic acid [3]
**Total types**	**25**	**25**	**26**	**26**	**26**	**26**	**26**		
**Total content**	**9265.02**	**10035.4**	**11708.85**	**14087.6**	**12192.95**	**15706.74**	**18253.53**		

Data are mean ± standard deviation. (n=3). Values with different letters in the same row are significantly different ($p < 0.05$). nd, not detected. -, not found.

The odor threshold and description have been taken from references.

Table S3. Volatile aroma compounds in Italian Riesling white wine with different maceration processes.

Content (μg/L)	Control	Cold maceration			Skin-contact fermentation			Threshold (μg/L)	Description
		24 h	48 h	72 h	25%	50%	100%		
**Alcohol**	**3691.96**	**4276.29**	**4650.63**	**5698.29**	**4929.2**	**5916.26**	**6661.73**		
Isoamyl alcohol	1966.14±254.31^{d}	2404.1±204.14^{cd}	2506.74±258.72^{cd}	2888.80±332.04^{bc}	3120.74±236.74^{ab}	3275.11±312.52^{ab}	3600.55±438.93^{a}	30,000 [1]	Harsh, stale, fusel odour [1]
1-Butanol	13.25±0.34^{de}	13.68±0.32^{e}	17.24±2.11^{c}	22.11±2.37^{b}	15.64±0.27^{cd}	18.31±1.34^{c}	29.91±1.33^{a}	15,000 [2]	Chemical, solvent, bitter [2]
1-Hexanol	418.12±57.63^{d}	472.70±102.74^{bcd}	584.58±174.36^{bcd}	735.52±137.49^{abc}	438.95±147.35^{cd}	760.87±157.34^{ab}	930.65±249.67^{a}	1,100 [2]	Herbaceous, grass, woody [2]
3,4-Hexanediol	10.04±1.24^{b}	13.77±2.14^{ab}	10.94±2.11^{b}	10.07±1.35^{b}	11.06±2.54^{b}	12.35±1.89^{ab}	15.18±1.57^{a}	-	-
Phenylethanol	1198.26±321.37^{c}	1280.29±224.37^{bc}	1433.22±237.24^{abc}	1913.34±398.34^{ab}	1266.27±264.35^{bc}	1755.59±445.43^{abc}	1966.15±357.34^{a}	14,000 [2]	Floral, rose [2]
Isobutanol	14.57±1.21^{a}	12.88±1.24^{a}	13.41±2.31^{a}	14.18±1.54^{a}	13.15±1.82^{a}	13.82±3.64^{a}	14.21±5.47^{a}	40,000 [2]	Medicinal, phenolic [2]
2,3-Butanediol	50.12±11.39^{abc}	54.55±12.36^{ab}	52.77±12.49^{abc}	70.84±16.24^{a}	35.60±2.34^{bc}	32.14±11.34^{c}	37.11±3.24^{bc}	15,000 [1]	Fruity, fresh [1]
3-Methyl-1-pentanol	20.15±3.21^{c}	22.18±4.87^{bc}	27.89±6.47^{bc}	37.45±9.88^{bc}	22.89±10.67^{bc}	42.27±12.34^{ab}	60.12±18.34^{a}	50,000 [1]	Vinous, herbaceous, cocoa [1]
1-Decanol	1.31±0.14^{e}	2.14±0.34^{de}	3.84±1. 12^{cd}	5.98±1.54^{ab}	4.90±0.57^{bc}	5.80±1.21^{bc}	7.85±1.11^{a}	400 [2]	Orange flowery, special fatty [2]
**Acid**	**172.84**	**195.03**	**262.14**	**290.11**	**202.33**	**176.18**	**232.86**		
Decanoic acid	102.24±21.37^{a}	124.21±24.94^{a}	139.00±23.73^{a}	132.40±45.37^{a}	54.31±12.34^{b}	47.39±6.41^{b}	49.71±9.67^{b}	1,000 [1]	Vinegar, animal, fatty, rancid, citrus, phenolic [1]
10-Undecenoic acid	10.34±2.24^{e}	11.64±2.71^{de}	17.82±6.37^{cde}	27.67±1.37^{bc}	22.35±2.97^{cd}	34.21±4.33^{ab}	41.39±11.98^{a}	-	-
Octanoic acid	2.11±0.42^{d}	2.54±0.64^{d}	8.30±2.33^{cd}	36.79±9.87^{b}	56.64±21.66^{a}	10.91±1.04^{cd}	24.84±5.34^{bc}	500 [1]	Goat rancid cheese, fatty, oily, acetic [1]
Lauric acid	22.34±3.74^{a}	19.68±2.78^{a}	24.96±7.51^{a}	23.71±3.20^{a}	22.24±3.47^{a}	24.12±3.27^{a}	26.43±2.55^{a}	1,000 [3]	Dried fruit, metallic taste [3]
2-Methylbutyric acid	35.14±3.41^{c}	34.21±4.69^{c}	68.39±22.31^{ab}	63.51±12.34^{ab}	44.31±6.34^{bc}	51.90±17.64^{abc}	77.78±18.61^{a}	33 [7]	Sweaty, cheese [7]

4-Methyloctanoic acid	0.67±0.01^{d}	0.94±0.12^{cd}	1.21±0.34^{cd}	1.35±0.34^{bc}	0.57±0.05^{d}	1.98±0.64^{ab}	2.37±0.39^{a}	-	-
**Ester**	**3493.66**	**4282.16**	**5036.4**	**5551.39**	**3656.76**	**4496.78**	**5208.1**		
Pentyl acetate	51.34±11.24^{d}	54.68±14.67^{cd}	53.14±21.03^{cd}	86.35±23.15^{bc}	71.21±8.34^{bcd}	96.41±24.31^{ab}	121.34±12.91^{a}	-	-
Isoamyl acetate	28.12±2.37^{d}	31.50±6.95^{d}	44.13±11.64^{cd}	63.12±19.60^{bc}	91.34±23.49^{ab}	97.26±12.31^{a}	107.38±16.34^{a}	30 [4]	Fresh, sweet, fruity [4]
Hexyl acetate	198.93±32.14^{c}	207.31±36.49^{c}	321.54±62.47^{c}	489.65±57.64^{b}	327.31±41.04^{c}	551.31±119.64^{ab}	639.21±103.01^{a}	670 [4]	Green, fruity, sweet, fatty, fresh [4]
Phenethyl acetate	455.83±102.34^{c}	489.31±64.31^{c}	631.44±103.42^{bc}	734.21±157.38^{ab}	432.12±84.61^{c}	632.34±52.34^{bc}	874.31±128.45^{a}e	1,800 [5]	Fruity, rose [5]
Heptyl acetate	48.12±6.54^{c}	52.47±12.67^{c}	54.31±17.43^{c}	57.82±12.37^{b}	49.78±12.01^{c}	53.12±9.48^{b}	57.42±12.67^{a}	1,400 [7]	Almond, pear [7]
Ethyl acetate	186.16±47.31^{d}	197.31±38.94^{d}	249.31±64.15^{cd}	347.14±68.37^{bc}	253.14±61.34^{cd}	431.28±67.61^{ab}	493.78±37.45^{a}	7,500 [4]	Caramel, sweet, fruit, buttery, pungent [4]
Ethyl butyrate	67.21±12.54^{c}	79.46±24.31^{c}	115.06±21.37^{bc}	128.47±6.47^{bc}	98.31±21.37^{bc}	157.70±35.46^{b}	254.30±66.34^{a}	20 [6]	Sour fruit, fruity, strawberry [6]
Ethyl octanoate	77.64±21.67^{c}	116.34±24.61^{c}	306.89±63.15^{b}	144.12±12.34^{c}	276.21±34.61^{b}	302.31±21.38^{b}	424.21±39.45^{a}	5 [1]	Fruity, sweet, waxy [1]
Ethyl decanoate	1987.21±234.87^{b}	2645.31±294.36^{a}	2834.64±349.51^{a}	2987.29±361.91^{a}	1681.74±212.36^{b}	1674.36±179.32^{b}	1634.61±236.54^{b}	200 [1]	Fruity, grape, pear, apple [1]
Ethyl hexanoate	364.78±64.38^{a}	381.72±21.37^{a}	397.71±87.06^{a}	474.10±64.35^{a}	336.42±41.37^{a}	458.39±34.16^{a}	551.27±243.97^{a}	80 [2]	Banana, green apple [2]
Methyl hydrogen azelate	23.45±2.37^{a}	21.64±2.75^{a}	22.17±4.32^{a}	22.14±1.38^{a}	27.31±4.39^{a}	26.34±1.35^{a}	25.21±6.34^{a}	-	-
Isopropyl palmitate	2.98±0.64^{c}	3.44±0.37^{c}	4.04±1.32^{bc}	8.66±2.34^{a}	5.53±0.34^{bc}	6.65±1.64^{ab}	6.35±1.34^{ab}	-	-
Dioctyl adipate	1.89±0.87^{cd}	1.67±0.70^{d}	2.02±0.12^{cd}	8.32±2.47^{b}	6.34±3.45^{bc}	9.31±2.48^{b}	18.71±3.22^{a}	-	-
**Aldehyde and Ketone**	**24.25**	**31.21**	**36.50**	**63.75**	**47.65**	**52.44**	**77.01**		
4-Methoxy-2,5-dimethylbenzaldehyde	4.39±0.85^{b}	7.45±1.33^{b}	6.31±3.15^{b}	10.47±1.34^{a}	nd	nd	nd	-	-
Dodecyl aldehyde	0.64±0.07^{c}	1.37±0.06^{c}	4.23±0.31^{bc}	7.45±3.48^{b}	12.39±2.46^{a}	nd	nd	-	-
**Terpene**	**71.34**	**80.87**	**119.33**	**149.62**	**118.45**	**183.07**	**233.42**		
Linalool	20.17±1.79^{c}	21.64±9.64^{c}	30.41±2.37^{bc}	37.54±3.78^{abc}	30.77±14.73^{ab}	45.37±12.45^{bc}	55.91±12.64^{a}	15 [1]	Rose, citrus [1]

Citronellol	40.81±12.36[a]	47.36±6.52[a]	59.31±19.74[a]	77.87±5.23[a]	54.31±17.37[a]	91.82±21.45[a]	118.34±24.98[a]	100 [2]	Rose [2]
Geraniol	10.36±2.38[c]	11.87±1.32[c]	29.61±8.37[bc]	34.21±11.47[b]	33.37±9.45[b]	45.88±14.36[ab]	59.17±14.37[a]	30 [1]	Rose, geranium [1]
**Others**	**37.08**	**50.48**	**53.36**	**70.01**	**49.54**	**62.98**	**80.21**		
Styrene	22.34±2.91[b]	35.63±8.61[ab]	37.63±9.87[ab]	57.42±15.32[a]	33.65±19.67[ab]	47.65±12.57[ab]	63.65±23.54[a]	250 [7]	Sweet [7]
2,4-Ditert-butylphenol	14.74±1.87[a]	14.85±2.64[a]	15.73±5.47[a]	12.59±1.33[a]	15.89±4.87[a]	15.33±2.47[a]	16.56±3.71[a]	200 [3]	Carbonic acid [3]
**Total types**	**35**	**35**	**35**	**35**	**34**	**33**	**33**		
**Total content**	**7471.91**	**8891.84**	**10129.94**	**11772.66**	**8966.76**	**10829.6**	**12405.98**		

Data are mean ± standard deviation. (n=3). Values with different letters in the same row are significantly different ($p < 0.05$). nd, not detected. -, not found.

The odor threshold and description have been taken from references.

Table S4. Recovery, repeatability and LOD of phenolic content by HPLC.

Content	Recovery (%)	Repeatability (%)	LOD (mg/L)
Gallic acid	89.23	1.67	0.026
Protocatechuic acid	92.11	1.55	0.058
Gentisic acid	90.58	1.88	0.1
Chlorogenic acid	89.92	2.26	0.14
Caffeic acid	93.25	1.44	0.046
p-Coumaric acid	93.03	1.90	0.022
Ferulic acid	92.23	1.72	0.027
C	85.19	1.57	0.011
EC	90.17	1.56	0.013
EgC	88.55	2.13	0.011
EgCg	85.28	2.21	0.011
Rutin	87.34	1.98	0.023
Quercitrin	91.43	1.83	0.036
Myricetin	90.56	2.37	0.025
Quercetin	86.72	1.92	0.016
Galangin	92.75	1.76	0.013

LOD, Limit of detection. C, catechin. EC, epicatechin. EgC, epigallocatechin. EgCg, epigallocatechin gallate.

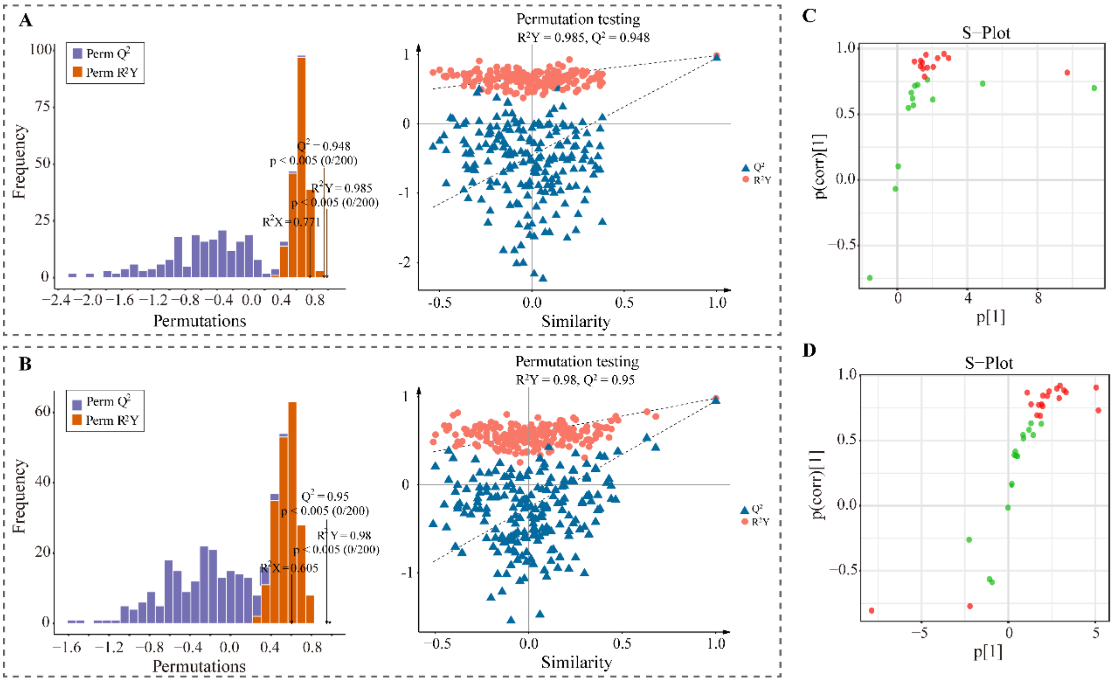

Figure S1. Model validation permutation test (A-B) and S-plot (C-D) of the volatile aroma compounds in Chardonnay (A and C) and Italian Riesling (B and D) white wines with different maceration processes.

References

[1] De Paolis, C.; Zava, A.; Paissoni, M.A.; Rio Segade, S.; Motta, G.; Skrab, D.; Beria D'Argentina, S.; Ferrero, L.; Giacosa, S.; Gerbi, V.; Rolle, L. Cold liquid stabulation: Impact on the phenolic, antioxidant, and aroma characteristics of wines from aroma-neutral white grape varieties. *Food Chem.* **2025,** *465*, 142058.

[2] Zhang, B.; Zhang, C.; Chen, J.; Zhao, C.; Du, Y.; Yang, Y.; Xie, X.; He, L.; Liu, S.; Shi, K. On-vine drying (passérillage) improves the quality of "Hutai No. 8" table grape wine: Focusing on phenolics, aromas, color and sensory attributes. *Food Chem.* **2025,** *463*, 141325.

[3] Yang, L.; Zhu, X.; Mao, Y.; Zhang, X.; Xu, B.; Yang, X. Effect of different inoculation strategies of mixed culture *Saccharomyces cerevisiae/Oenococcus oeni* on the aroma quality of Chardonnay wine. *Food Res. Int.* **2024,** *190*, 114636.

[4] Alti-Palacios, L.; Martinez, J.; Teixeira, J.A.C.; Camara, J.S.; Perestrelo, R. Influence of cold pre-fermentation maceration on the volatilomic pattern and aroma of white wines. *Foods* **2023,** *12*, 1135.

[5] Cai, J.; Zhu, B.; Wang, Y.; Lu, L.; Lan, Y.; Reeves, M.J.; Duan, C. Influence of pre-fermentation cold maceration treatment on aroma compounds of Cabernet Sauvignon wines fermented in different industrial scale fermenters. *Food Chem.* **2014,** *154*, 217-229.

[6] Li, N.; Li, G.; Guan, X.; Li, A.; Tao, Y. Volatile aroma compound-based decoding and prediction of sweet berry aromas in dry red wine. *Food Chem.* **2025,** *463*, 141248.

[7] Guo, Y.; Gao, D.; Wang, Z.; He, Y.; Wang, S.; Wang, H.; Shi, H. A comparative study of the flavor characteristics and active ingredients of fermented and blended orange wines based on sensory omics and non-targeted metabolomics. *LWT* **2025,** *223*, 117719.
